# Knowledge, Attitude, and Practices Towards COVID-19 Among Pregnant and Postpartum Women in Rural Bangladesh: A Cross-Sectional Study

**DOI:** 10.1007/s10995-024-03900-y

**Published:** 2024-01-31

**Authors:** Gayani Gunawardhana, S. M. Rokonuzzaman, Sajia Islam, Neeloy Alarm, Tarana E Ferdous, Elizabeth K Kirkwood, Camille Raynes-Greenow, Sk Masum Billah

**Affiliations:** 1 Sydney School of Public Health, The University of Sydney, Camperdown, Australia; 2 Maternal and Child Health Division, ICDDR, Dhaka, Bangladesh

**Keywords:** COVID-19, Pregnant women, Preventive behaviours

## Abstract

**Objective:**

To identify knowledge, attitudes, and practices toward COVID-19 among pregnant and postpartum women in rural Bangladesh, and to assess any association with antenatal care attendance (ANC).

**Methods:**

This cross-sectional study was conducted in Northern Bangladesh’s Sherpur district with a sample of pregnant and post-partum women enrolled in ‘Poriborton’, a community-based cluster Randomised Controlled Trial. Knowledge, attitude, and practices toward COVID-19, and any association with antenatal care practices were assessed through face-to-face interviews using a structured questionnaire. Composite measures for knowledge, attitude, and practices of COVID-19 were generated. Specific knowledge on COVID-19 and the association of antenatal care were presented as descriptive statistics. An adjusted odds ratio was used to examine the association between categorical variables.

**Results:**

Out of 4835 women, 34.0% correctly identified five or more COVID-19 symptoms, 31.8% knew three or more modes of coronavirus transmission, and 57.0% knew five or more preventive measures. Most (90.1%) had a “more positive attitude to testing” and 65.1% reported adequate practice of preventive measures. Women with adequate knowledge of COVID-19 were more likely to report both a positive attitude to testing (OR:2.96; 95% CI: 1.38–6.37) and adopt adequate practices of preventive measures (OR: 4.30;95% CI: 2.90–6.36). Education and being employed influenced KAP related to COVID-19. Only 14.9% were satisfied with their knowledge of COVID-19. Television was the main source of COVID-19-related specific information.

**Conclusions:**

We found that improved knowledge was associated with positive attitudes and behaviours that lead to the adoption of preventive measures. There was no association with ANC practices as attendance was very low in this region. The findings could be utilised to develop communication strategies for future public health emergencies in similar settings.

## Significance

Women with increased knowledge of COVID-19, led to more receptive attitudes and behaviors to reduce the risk of corona virus infection. This was modified by sociodemographic factors in this rural Bangladeshi community. These findings can be used to develop more targeted communication strategies for future public health emergencies.

## Introduction

The World Health Organization (WHO) on March 11, 2020, declared the outbreak of the novel coronavirus (COVID-19) a global pandemic (Cucinotta & Vanelli, 2020). Pregnant women were more likely to have severe COVID-19 disease compared to women who were not pregnant (Kotlar et al., 2021), and COVID-19 increased the risk of maternal morbidity, mortality, and neonatal complications (Villar et al., 2021). The impact of coronavirus infection also indirectly affected pregnant women through changes in healthcare provision, and other broader social and economic changes (Kotlar et al., 2021). Some of these changes were facilitated through the control measures implemented such as transport restrictions and lockdowns, which resulted in delays in accessing healthcare facilities for obstetric emergencies (Aranda et al., 2022). The rapid spread of the disease and the unpreparedness of the health sector affected antenatal and postnatal care, which resulted in increased home births and maternal morbidity and mortality (De Carvalho-Sauer et al., 2021; Figliozzi & Unnikrishnan, 2021; Calvert et al., 2021). Antenatal and postnatal care seeking was also reduced worldwide ( Naqvi et al., 2022) resulting in increased maternal and neonatal morbidity and mortality (Calvert et al., 2021).

Many preventative interventions utilise behaviour change communication to reduce the risk of the spread of COVID-19. According to the knowledge, attitude, and practice theory, human behaviour changes in three steps: acquiring knowledge, generating attitudes or beliefs, and forming practices or behaviours (Kim et al., 1969; Alsaleh et al., 2023; Wang et al., 2020). People who acquire adequate knowledge and a positive attitude are more likely to adopt preventative behaviours (Brügger & Höchli, 2019; Mat Dawi et al., 2021). Hence, effective, and appropriate communication is needed to support behaviour change and adherence to infection control practices such as the correct wearing of masks and proper handwashing with soap and water (Cucinotta & Vanelli, 2020; Ning et al., 2020).

This study aimed to identify knowledge, attitudes, and practices (KAP) towards COVID-19, and assess the association of the pandemic with antenatal care among pregnant mothers in a rural area of Bangladesh. This study will help inform the development of behaviour change communication strategies for future public health emergencies in other similar settings.

## Method

This study was embedded into an ongoing community-based cluster randomized controlled trial named “Poriborton: The CHANge Trial” (Raynes-Greenow et al., 2022). The trial investigated reduced exposure to household air pollution through the provision of liquid petroleum gas cooking compared to usual cooking to assess the impact on perinatal outcomes in pregnant women in Sherpur, Mymensingh division, Bangladesh. Pregnant women who were permanent residents aged between 15 and 49 years and enrolled in the trial were invited to participate in this sub-study.

We used a cross-sectional design utilising the baseline data with additional primary data collected using a separate interviewer-administered questionnaire regarding the KAP of COVID-19, and antenatal care (ANC) use. The questionnaire was developed based on the WHO survey tool guidance, 2020 (WHO, 2020), and with expert opinion. Bilingual experts translated the survey tool into Bangla, and pilot-tested it and adopted it according to the Bangladesh context. The final version included 45 fixed responses and open-ended questions to assess COVID-19 KAP, risk perception, sources of information, and the association with antenatal care practices. Data collection was conducted between November 2020 to August 2021.

### Data Analysis

For the analysis, based on previous research, we calculated a composite measure of adequate knowledge, that was achieved if participants were able to correctly identify five or more symptoms out of a possible thirteen, three or more correct transmission modes out of nine, and five or more preventive methods out of twenty, and identifying early detection and symptomatic treatment to help recover from COVID-19 (Naqvi et al., 2022). A “more positive attitude to testing” was defined as those participants who agreed with the statements “All people with symptoms should do a COVID-19 test is very important” and “Go for testing and care seeking if develop symptoms”. We defined adequate practice towards COVID-19 with participants who regularly practiced wearing face masks and proper hand washing.

Demographic characteristics of the participants and their KAP of COVID-19 are presented as frequencies and percentages. Multiple logistic regression analysis was performed. An adjusted odds ratio was used to examine associations between categorical variables. Data analysis was performed using IBM Statistical Package for the Social Sciences (SPSS) -version 28 (IBM, 2021).

#### Ethical Approvals

Ethical approval was obtained from the International Centre for Diarrhoeal Disease Research, Bangladesh (No.PR-17,103). All women who participated provided informed consent for participation and data collection.

## Results

Of the 4944 pregnant and post-partum women from the original list of enrolled women in the trial, 4835 were included. Most were pregnant (n = 2949, 61%) and the remainder were post-partum women (n = 1886, 39%). Participants were predominately aged between 20 and 35 (n = 3746, 77.5%). Most of the study participants had attended some school (n = 4324, 89.4%), and only 6.8% (n = 329) were in paid employment (Table 1).

**Table 1:** Socio-demographic characteristics of the pregnant and postpartum women in Mymensingh division, Bangladesh

Characteristic	Number (n = 4835)	Percentage (%)
*Age (years)*		
<20	988	20.4
20–35	3746	77.5
≥ 36	101	2.1
*Marital status*		
Married	4820	99.7
Unmarried	15	0.3
*Education level*		
No formal education	511	10.6
Primary	1548	32.0
Secondary	2127	44.0
Tertiary	649	13.4
*Employment status*		
Employed	329	6.8
No formal employment	4506	93.2
*Parity*		
0	1530	31.6
1–4	3262	67.5
≥ 5	43	0.9

Nearly one-third of women (34.0%, n = 1646) correctly identified five or more symptoms of COVID-19 out of thirteen and 31.8% (n = 1537) of women identified three or more transmission modes correctly out of nine. More than half 57.0% (n = 2758) of women identified five or more preventive measures correctly out of twenty. Of all women who participated, 42.9% (n = 2074) knew that ‘early detection and symptomatic treatment’ can help the infected person recover from COVID-19. Adequate knowledge of overall aspects of COVID-19 (symptoms, mode of transmission, preventive measures, and treatment) was reported by 6.3% (n = 306) of women (Table 2). The main source of information on the mode of transmission of coronavirus was ‘television’ (73.2%, n = 3541).

Specific and in-depth knowledge of COVID-19 was assessed, and nearly half (48.9%) of respondents correctly identified the incubation period was up to 14 days. The main source of information on this was ‘television’ (n = 3649, 75.5%). Only 32.1% of the study participants knew that the elderly is a high-risk group with an increased risk of morbidity and mortality (Yanez et al., 2020). A high proportion (n = 3858, 79.8%) of study participants incorrectly identified that ‘cough’ is a symptom that needs hospital admission, while only 42.2% (n = 2041) correctly identified that difficulty in breathing requires hospital admission. Half of the participants (n = 2587, 53.5%) thought a blood test was needed to identify COVID-19. Most of the study participants (n = 3937, 81.4%) had thought that children can be infected with coronavirus from breastfeeding by an infected mother and nearly half mentioned that the source of this information was television (n = 2296, 47.5%). Out of all study participants, television was stated as the most trustworthy (n = 3573, 73.9%) source of information.

Adequate knowledge of COVID-19 increased with the level of education, women who had secondary (aOR:2.74: 95% CI:1.48–5.07) or tertiary (OR:5.65; 95% CI:2.95–10.84) education had more adequate knowledge compared to women with no formal education. Employed women (aOR:1.56; 95% CI:1.01–2.25) had better knowledge than women (Table 3) who were not in formal employment. Self-rated knowledge of COVID-19 was considered ‘poor’ by 30.3% (n = 1463), and ‘very poor’ by 9.5% (n = 460). Only 14.9% (n = 719) were satisfied with their knowledge of COVID-19.

**Table 2:** Composite measures of KAP toward COVID-19 among pregnant and post-partum women in Mymensingh Division, Bangladesh

Characteristics	Number (n = 4835)	Percentage (%)
*Adequate knowledge-a composite measure*	306	6.3
Named ≥ 5 symptoms correctly	1646	34.0
Named ≥ 3 modes of transmission correctly	1537	31.8
Named ≥ 5 preventive measures correctly	2758	57.0
Identified ‘Early detection and symptomatic treatment’ can help the infected person to recover	2074	42.9
*Positive attitude- a composite measure*	4356	90.1
Mentioned ‘Testing for COVID-19 is very important’	4507	93.2
Mentioned ‘If they developed symptoms, they would go to a healthcare center for testing or care-seeking’	4578	94.7
*Adequate practice of preventive measures –* *a composite measure*	3148	65.1
Regularly wash hands to prevent COVID-19	3783	78.2
Regular use of face masks to prevent COVID-19	3517	72.7

Most of the study participants (n = 4356, 90.1%) had a ‘positive attitude to testing’ for COVID-19 and considered that testing was very important (n = 4507, 93.2%) and should be done when symptomatic. Most respondents (n = 4578, 94.7%) reported that they will go for testing and seek healthcare if they developed symptoms. A ‘more positive attitude to testing’ for COVID-19 was lower among women aged between 20 and 35 years compared to women aged below 20 years (aOR:0.66; 95% CI:0.46–0.96), and higher among women who are employed compared to women who were not in formal employment (OR: 1.42; 95% CI:0.90–2.23), and among women with primary (OR:1.58; 95% CI:1.20–2.08), secondary (OR = 2.68; 95% CI = 2.02–3.57), or tertiary education (OR:3.45; 95% CI: 2.27–5.24) compared to women with no formal education. A quarter of women (n = 1228, 25.4%) reported that the testing procedure for COVID-19 was uncomfortable.

**Table 3:** Association between selected socio-demographic factors with KAP towards COVID-19 among pregnant and recent post-partum women in Mymensingh Division, Bangladesh

Variable	Knowledge			Attitude			Practice		
	Adequate N (%)	Inadequate N (%)	aOR (95% CI)	More positive N (%)	Less positive N (%)	aOR (95% CI)	Adequate N (%)	Inadequate N (%)	aOR (95% CI)
*Age (years)*									
< 20	51 (5.2)	937(94.8)	Ref	917 (92.8)	71 (7.2)	Ref	615 (62.2)	373 (37.8)	Ref
20–35	249 (6.6)	3497(93.4)	0.84(0.56–1.26)	3354 (89.5)	392 (10.5)	0.66(0.46–0.96)	2478 (66.2)	1268 (33.8)	1.01(0.82–1.25)
> 35	6(5.9)	95(94.1)	0.39(0.23–1.77)	87 (86.1)	14 (13.9)	0.74(0.29–1.87)	56 (55.5)	45 (44.5)	0.79 (0.44–1.41)
*Education level at school*									
Primary	63(4.1)	1485(95.9)	1.65(0.87–3.12)	1360 (87.9)	188 (12.1)	1.58(1.20–2.08)	931 (60.1)	617 (39.9)	1.21(0.98–1.50)
Secondary	141(6.6)	1986(93.4)	2.74(1.48–5.07)	1971 (92.7)	156 (7.3)	2.68(2.02–3.57)	1433 (67.4)	694 (32.6)	1.54(1.29–1.97)
Tertiary	90(13.9)	559(86.1)	5.66(2.95–10.84)	611 (94.1)	38 (5.9)	3.45(2.27–5.24)	515 (79.4)	134 (20.6)	2.57(1.93–3.42)
Not attended	12(2.3)	499 (97.7)	Ref	416 (81.4)	95 (18.6)	Ref	270 (52.8)	241 (47.2)	Ref
*Employment status*									
Employed	40(12.2)	289(87.8)	156(1.01–2.25)	307 (93.3)	22 (6.7)	1.42(0.90–2.23)	266 (80.6)	63 (19.1)	2.03(1.51–2.71)
Unemployed	266(5.9)	4240(94.1)	Ref	4051 (89.9)	455 (10.1)	Ref	2883 (64.0)	1623 (36.0)	Ref
*Marital status*									
Married	306 (3.6)	4514 (93.7)		4393 (91.1)	477 (9.9)		3141 (65.2)	1679 (34.8)	1.83(0.65–5.15)
Unmarried	0	15 (100.0)		15 (100.0)	0		8 (53.3)	7 (46.7)	
*Parity*									
0	101(6.6)	1429 (93.4)	1.69(0.22–13.39)	1001(65.4)	529(34.6)	1.01 (0.41–2.51)	1410(922)	120(7.8)	1.31 (0.67–2.54)
1–4	204 (6.3)	3058(93.7)	1.91(0.24–14.75)	2127 (65.2)	1135 (34.8)	1.08 (0.45–2.58)	2912 (89.3)	350 (10.7)	1.43 (0.75–2.73)
> 5	1(2.3)	42(97.7)	Ref	21 (48.8)	22 (51.2)	Ref	36 (83.7)	7 (16.3)	Ref
*Knowledge*									
adequate				604 (97.6)	15 (2.4)	2.96(1.38–6.37)	528 (85.3)	91 (14.7)	4.30 (2.90–6.36)
Inadequate				3754 (89.0)	462 (11.0)	Ref	2621 (62.2)	1595 (37.8)	Ref
*Attitude*									
More positive							2968 (68.1)	1390 (31.9)	3.07 (2.51–3.75)
Less positive							181(37.9)	296 (62.1)	Ref

**Table 4:** Frequency distribution of selected variables on KAP toward COVID-19 among pregnant and postpartum women in Mymensingh division, Bangladesh

Characteristics	Number (n = 4835)	Percentage (%)
*Mentioned the following as symptoms of COVID-19*		
Fever	4659	96.4
Shortness of breath	2179	45.1
Sore Throat	3050	63.1
Cough	4362	90.2
*Identified the following as the mode of transmission*		
Respiratory droplets from infected individuals	3750	77.6
Physical contact with an infected person without protection	1412	29.2
Touching the dead body of an infected person without protection	132	2.7
Living in the same building with an infected person	925	19.1
*Knowledge of high-risk age groups*		
Children, up to 17yrs	334	6.9
Young people (18 to 34 years)	36	0.7
Middle-aged (35–50 years)	293	6.1
Elderly (51 + years)	1551	32.1
*Knowledge about the treatment of COVID-19*		
Currently, there is no effective cure	1563	32.3
Early detection and symptomatic treatment can help to recover	2074	42.9
COVID-specific drugs are available	1675	34.6
*Knowledge of preventive measures*		
Maintaining physical distancing	3348	69.2
Maintaining personal hygiene	2916	60.3
Handwashing frequently with soap	2921	60.4
Wearing masks	2716	56.2
*Attitude towards if someone develops symptoms of COVID-19*		
Send to nearest govt hospital for testing	3991	82.5
Call the hotline number for testing	625	12.9
Consult a physician/health care provider	695	14.4
*Attitude towards if someone is diagnosed with COVID-19*		
Isolate from others	4171	86.3
Immediate hospitalization referral in case of severe condition	623	12.9
Taking medication as prescribed by doctors	1850	38.3
*Attitude toward the test*		
It is an easy and simple procedure	1314	27.2
It is a long procedure	237	4.9
Very tough to be tested	1228	25.4
This is an expensive test	221	4.6
*Practice - Out of all who regularly washed their hands (n = 3783)*		
Wash hands with soap and water	3694	97.7
Wash hands for more than 20 s	1804	47.6
*Practice- Out of all who regularly wear face masks (n = 3571)*		
Wear it while going outside	3501	98.0
Wear it while talking to other people	118	3.3

Out of all study participants, almost three-quarters (n = 3517, 72.7%) used facemasks regularly and more than three-quarters (n = 3783, 78.2%) washed their hands regularly. The composite measure of an adequate practice of preventive measures (both regular use of face masks and hand washing) was reported in 65.1% (n = 3148) of women (Table 4). Higher levels of educational attainment and employment were associated with the adequate practice: those with tertiary education (OR:2.57; 95% CI:1.93–3.42) compared to women with no formal education, and those who were employed compared to women who were not in formal employment (OR:2.03; 95% CI:1.51–2.71) were most likely to adopt adequate practices. Adequate knowledge of COVID-19 was also associated with a ‘more positive attitude to test’ for COVID-19 (OR:2.96; 95% CI:1.38–6.37) and adequate practice of preventive measures (OR:4.30; 95% CI:2.90–6.36).

Most of the currently pregnant women (n = 2526 out of 2949, 85.6%) did not receive routine antenatal care (ANC) from any formal provider. The main reason reported was that they did not consider ANC necessary during pregnancy (n = 2311 out of 2526, 91.4%), with only a minority (n = 13 out of 2526, 0.5%) not seeking ANC due to fear of being infected with COVID-19. Another 0.8% (n = 22 out of 2526) of women did not seek ANC as they doubted that the ANC care providers were present to provide care during the pandemic. A small number of women (n = 41 out of 2526) (1.6%) did not seek ANC due to a lack of transportation and lack of money during the pandemic. Almost all women (97.6%) who participated in the study mentioned that their intention to seek ANC was not disrupted due to the COVID-19 pandemic.

## Discussion

### Main Findings

We found that education and being employed influenced KAP related to COVID-19 among pregnant and postpartum women. Our study highlighted those women with adequate knowledge of COVID-19 (symptoms, mode of transmission, preventive measures, and treatments) had a ‘more positive attitude of testing’ and adequate practice of preventive measures for COVID-19, which supports the theory of KAP (Kim et al., 1969; Alsaleh et al., 2023; Wang et al., 2020). More than one-third of the women were not satisfied with their knowledge of COVID-19. Specific knowledge on some aspects of COVID-19 (type of test used, symptoms need hospital admission) was poor. Nearly three-quarters of women mentioned ‘television’ as their main source of COVID-19 information.

### Strengths and Limitation

The strengths of this study were that we collected prospective data with a large sample size. This community-based study utilised face-to-face interviews with women of rural areas to facilitate the identification of knowledge, attitude, and practice gaps in a rural and relatively low socio-economic population. Whereas most research on COVID-19 data collection was online, limiting the availability of data to educated and economically wealthy populations. Well-trained, experienced data collectors familiar with the mothers who collected the data led to minimizing the observation bias. We were unable to validate the data collection tool due to the prevailing pandemic restrictions at the time of this research is a limitation of this study. We also acknowledge that the cut off value for adequacy of knowledge, attitude and practices are arbitrary and best values were taken with the expert opinions.

## Interpretation

A substantial proportion of women in the study population were less than 20 years old and uneducated. According to the Bangladesh Demographic and Health Survey (BDHS) 2017, low literacy in ever-married women is high and 23.9% of ever-married women between the ages of 30–34 are illiterate, and the percentage increased with age (NIPORT, 2020). Any information aimed at these women needs to consider their low literacy levels and adapt behaviour change communication messaging accordingly (Lee et al., 2021).

Despite the low levels of literacy, we found that a higher percentage of women in our study had adequate knowledge of preventative measures compared to other similar studies with pregnant women such as an online survey done in Wuhan in March 2020 (Ding et al., 2021), The comparatively adequate knowledge of preventive measures in this population could be a result of mass media, especially the media’s focus on COVID-19 symptoms and preventive measures at the time of the data collection. Selection of the correct channel of dissemination of the information is also important. In our study, nearly three-quarters of women mentioned ‘television’ as their source of information.

In our study, most of the women described that their infants could be infected with COVID-19 through breastfeeding. In a scoping review done in 2021, the impact of COVID-19 on maternal health identified that vertical transmission of coronavirus is unlikely (Kotlar et al., 2021). This knowledge gap could be attributed to the time our research was conducted between the end of 2020 and the first half of 2021. There was continued uncertainty about vertical transmission during that time and the widespread notion that mothers with COVID-19 should not breastfeed (Vilar-Compte et al., 2021). More than half (51.3%) of pregnant mothers in a 2021 KAP study in Bangkok, Thailand revealed that they would not breastfeed if infected with COVID-19 (Kunno et al., 2022). Breastfeeding is critical to infants, and breastfeeding practices are sensitive to negative messages (Vilar-Compte et al., 2021; Foss & Southwell, 2006). Thus, to curtail the negative effect of breastfeeding practices, counselling is needed for women, including their families, that the benefit of breastfeeding outweighs the potential risk for coronavirus transmission to infants (Vilar-Compte et al., 2021; Walker et al., 2022; Latorre et al., 2021; Negin et al., 2016). The effective delivery of correct messages regarding breastfeeding could be achieved fast through the channel of ‘television’. In our study, nearly half of the women mentioned the main source of information on this was ‘television’ and three-quarters mentioned it as the most trustworthy information source. Mass media campaigns help prevent negative changes in health-related behaviours across a large population (Wakefield et al., 2010).

More than a third of the women in the study were not satisfied with their knowledge of COVID-19. Knowledge and awareness of the diseases are important parameters for the adaptation of protective measures that minimize the exposure risk of the illness (Mat Dawi et al., 2021; Ning et al., 2020; Vilar-Compte et al., 2021).

Most participants had a positive attitude to testing in this study. However, more than a quarter mentioned that testing for COVID-19 was uncomfortable. We did not collect data on whether women had been tested and therefore cannot speculate whether this perception would influence testing practices. It should also be noted that testing was relatively expensive in this setting and that the data were collected during the 2nd wave of COVID-19 when the infection rate was very high (Bari & Sultana, 2021).

A study in seven low and middle-income countries revealed a significant decline in the use of maternal health services in all seven countries except Malawi during the COVID-19 pandemic in 2020 (Naqvi et al., 2022). A scoping review of 95 publications reported that antenatal care visits decreased, healthcare infrastructure was strained, and potentially harmful policies were implemented with little evidence due to the COVID-19 pandemic. Compared to studies in other countries, the association of COVID-19 and ANC was low in this study area. The reason for this low association is due to persistent low coverage of ANC attendance. In our study, most women did not consider that ANC was necessary during pregnancy and, almost all mentioned that their practices to attend ANC was not disrupted due to COVID-19. According to the 2017 BDHS, only 38.1% of women who birthed during the previous three years in Mymensingh division (43.7% nationally) had received more than four of the recommended antenatal visits with at least one visit with a medically trained person, and 72.2% of women in the Mymensingh division (81.9% nationally) received at least one ANC from a medically trained provider (NIPORT, 2020; NIPORT & ICF, 2023). Moreover, Sherpur is the lowest-performing district among the four districts in the Mymensingh division in terms of ANC by medically trained providers (NIPORT, icddr,b and MEASURE Evaluation, 2019), and as such, a lower coverage of ANC indicators compared to the estimates for the Mymensingh division is expected.

These findings of ANC coverage in the BDHS demonstrate disparities in the uptake of health services by pregnant women in Bangladesh. A literature-based analysis of maternal healthcare in rural Bangladesh revealed a significant difference in rich-poor, urban-rural, and other socio-demographic factors in terms of access to and utilization of maternal care (Noor & Rushdi Saif, 2021).

## Conclusion and Recommendation

Knowledge of recently emerged diseases like COVID-19 is continuously evolving. Updated knowledge through reliable information facilitates the development of favourable attitudes and preventive behaviours in communities. Our findings demonstrated that being educated and employed positively influenced KAP.

To implement effective containment methods to curtail future pandemics in these remote areas with a high percentage of women with no formal education, special attention should be focused on delivering the information in a simple and culturally acceptable method. A mitigation plan with strategies to communicate, accurate health information timely is a must in managing future public health emergencies. According to the results, delivery of health information through ‘television’ would be a better option to cover a large population on time during pandemics.

Our research findings highlighted the underutilization of already available ANC facilities. We suggest a mass-scale community awareness program on the importance of receiving minimal routine ANC coverage to reduce neonatal and maternal mortality and morbidity.

## Data Availability

The dataset during and/or analyzed during the current study is available from the corresponding author at a reasonable request.
